# Supplementary material for: Comprehensive Characterization of Human Lung Large Cell Carcinoma Identifies Transcriptomic Signatures with Potential Implications in Response to Immunotherapy
Source: J Clin Med. 2022 Mar 9;11(6):1500. doi: 10.3390/jcm11061500 (PMC8953709; doi:10.3390/jcm11061500)
Supplement: Supplementary file 1 [file jcm-11-01500-s001.zip › Figure S1.pdf]

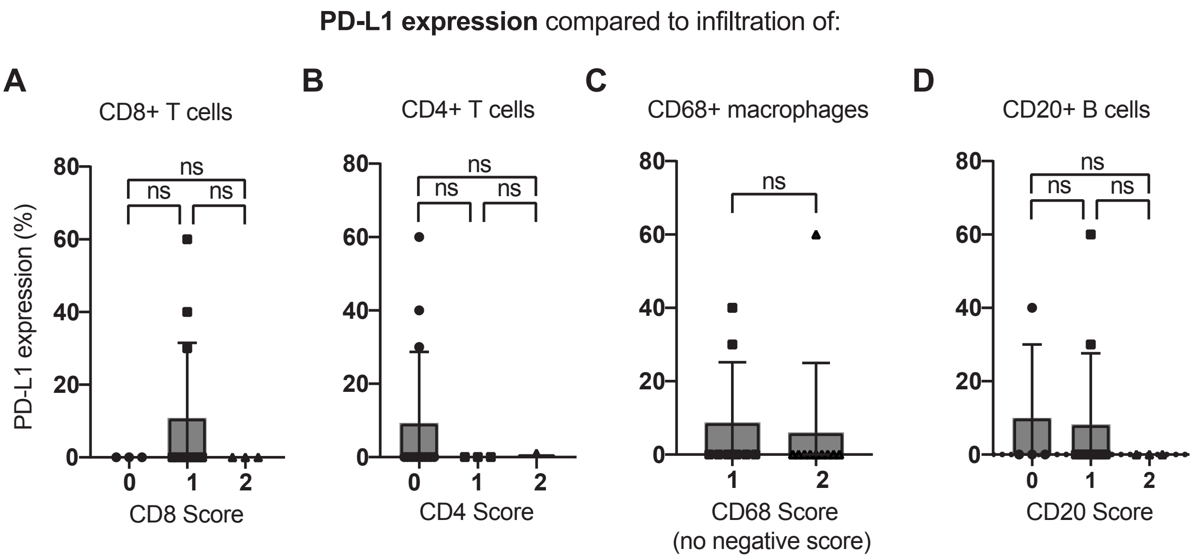

Figure S1. PD-L1 expression and level of infiltration of the evaluated immune cell populations.

Correlation between PD-L1 expression and immune infiltration from (A) T CD8+, (B) T CD4+, (C), CD68+ macrophages and (D) CD20+ B cells. Score 0: <1%, negative; Score 1: 1-10%, low expression; Score 2: >10%, high expression. No sample had a Score 0 for CD68+ macrophages. Staining ≥1% was considered to represent positivity for PD-L1.
