# Supplementary material for: Comprehensive Characterization of Human Lung Large Cell Carcinoma Identifies Transcriptomic Signatures with Potential Implications in Response to Immunotherapy
Source: J Clin Med. 2022 Mar 9;11(6):1500. doi: 10.3390/jcm11061500 (PMC8953709; doi:10.3390/jcm11061500)
Supplement: Supplementary file 1 [file jcm-11-01500-s001.zip › Figure S2.pdf]

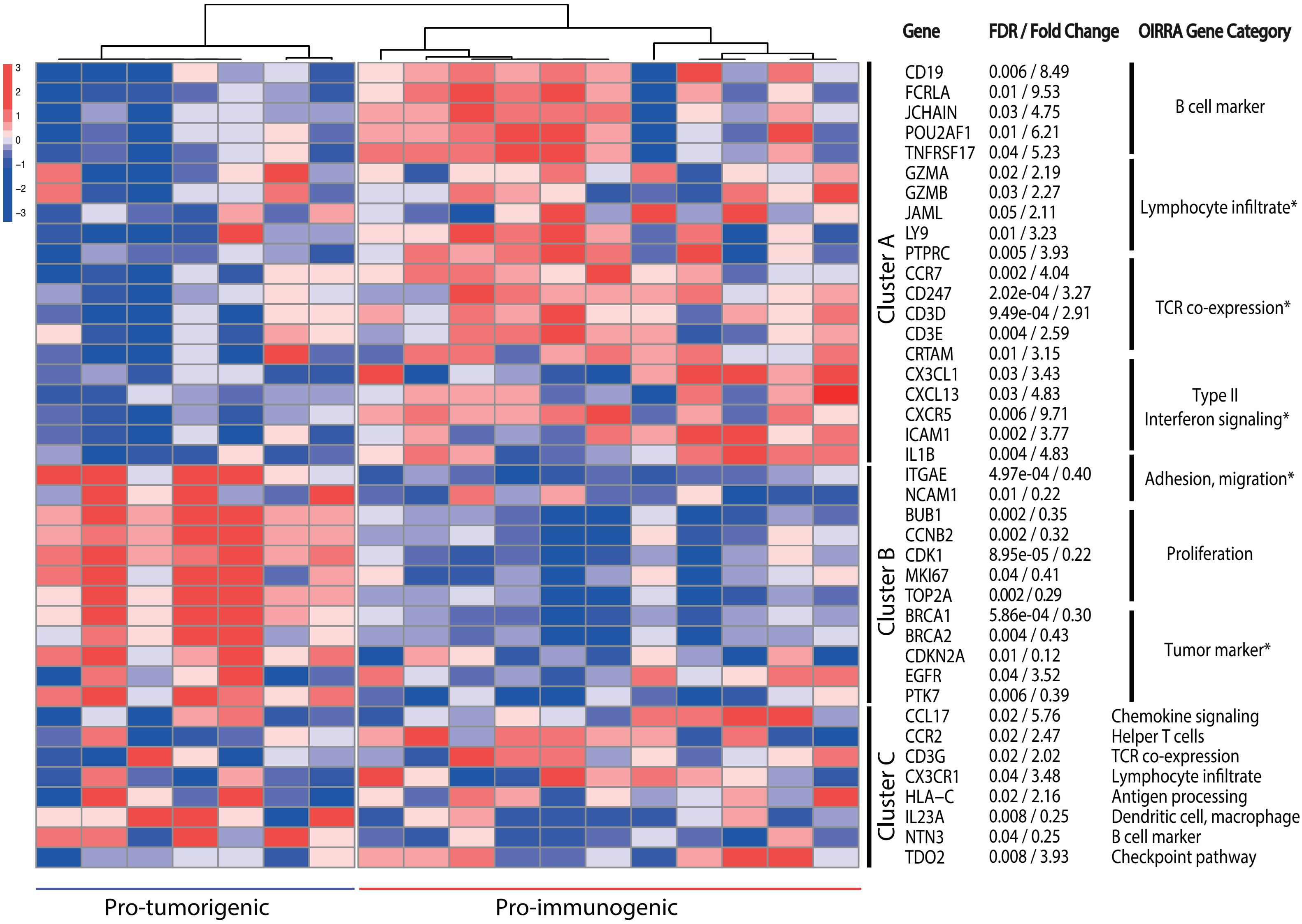

Figure S2. Representative genes of each cluster and respective OIRRA gene categories.

Heatmap of expression of representative genes of gene clusters A, B and C. Only differentially expressed genes were selected. Differential expression analysis was performed directionally, comparing pro-immunogenic tumors vs. pro-tumorigenic tumors. For each gene, FDR and Fold Change are provided. Specific OIRRA gene categories are specified. For gene clusters A and B statistically significant (*) and over-represented OIRRA gene categories were prioritized. For cluster C, all differentially expressed genes were selected.
