# Supplementary material for: Comprehensive Characterization of Human Lung Large Cell Carcinoma Identifies Transcriptomic Signatures with Potential Implications in Response to Immunotherapy
Source: J Clin Med. 2022 Mar 9;11(6):1500. doi: 10.3390/jcm11061500 (PMC8953709; doi:10.3390/jcm11061500)
Supplement: Supplementary file 1 [file jcm-11-01500-s001.zip › Figure S3_reviewed.pdf]

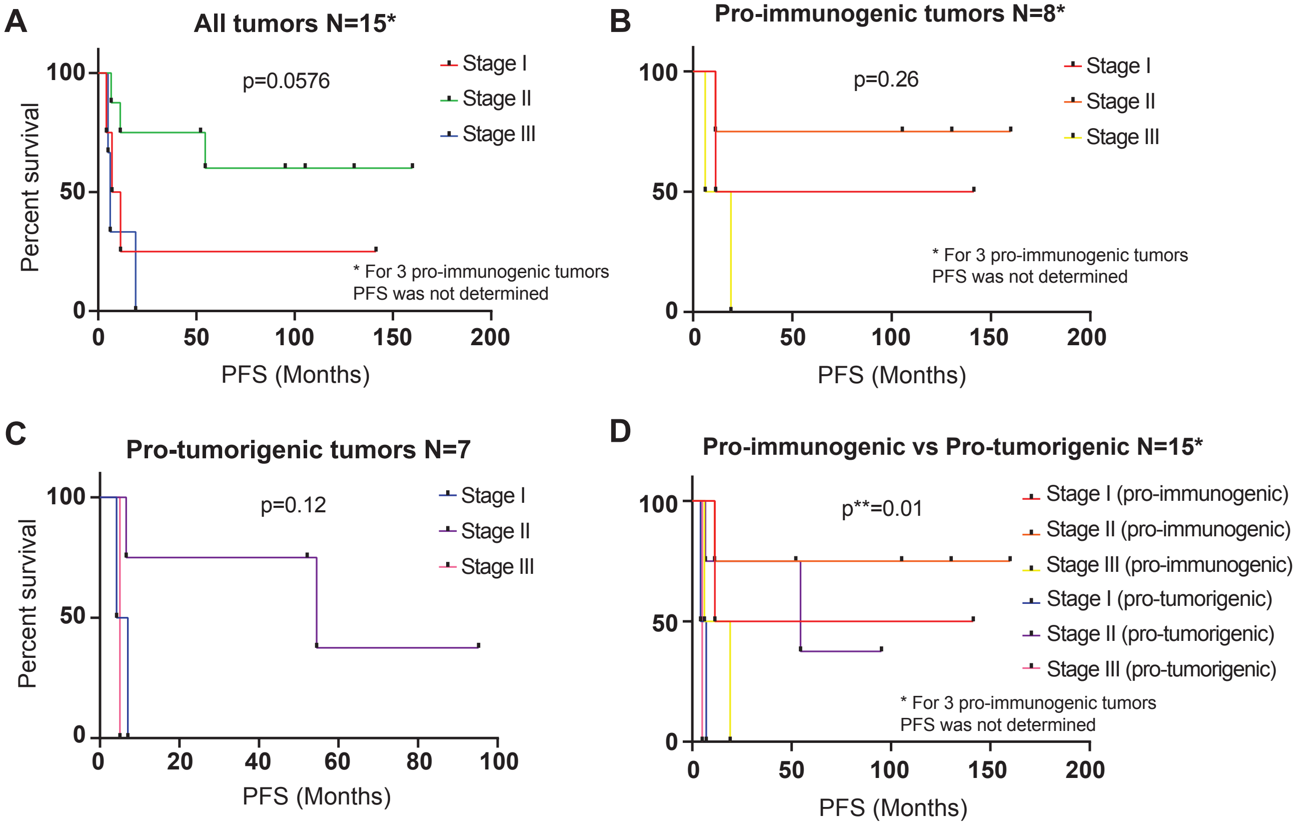

Figure S3. Progression-free survival analysis of the LCC tumors according to their stage and transcriptomic subgroup. Kaplan-Meier analysis of progression-free survival (PFS) according to stage in (A) all LCC tumors, (B) the pro-immunogenic tumors, (C), the pro-tumorigenic tumors and (D) all tumors divided by their transcriptomic subgroup.
